# Enhancement of the Thermostability of *Microbacterium* Esterase by Combinatorial Rational Design

**DOI:** 10.3390/molecules29245839

**Published:** 2024-12-11

**Authors:** Wenyu Peng, Xiaomei Wu, Baodi Ma, Yi Xu

**Affiliations:** School of Chemical and Environmental Engineering, Shanghai Institute of Technology, Shanghai 201418, Chinawuxiaomei@sit.edu.cn (X.W.)

**Keywords:** biotin, rational design, esterase, stability, protein engineering

## Abstract

The esterase EstSIT01 from *Microbacterium* can catalyze the asymmetric hydrolysis of *meso*-dimethyl ester to produce the crucial chiral intermediate (4*S*, 5*R*)-hemimethyl ester for *d*-biotin synthesis. Despite its high yields and stereoselectivity, the low thermostability of EstSIT01 limits its practical application. Herein, two kinds of rational strategies were combined to enhance the thermostability of EstSIT01. Based on the Surface Residue Substitution (SRS) method, two variants (G215A and G316A) with improved thermostability and one mutant (G293A) with superior activity were identified from nine candidates. According to the Consensus Mutation method, two mutants (E301P and A332P) with enhanced thermostability were identified from six candidates. However, the combined mutation failed to yield mutants surpassing the best single mutant, E301P, in terms of thermostability. The combined mutant E301P/G215A and E301P/G215A/G293A exhibited a slight enhancement in enzyme activity relative to E301P, while also exhibiting improved thermostability compared to the wild-type EstSIT01. Compared with the wild-type esterase, the thermal inactivation half-lives (*t*_1/2_) of mutant E301P were enhanced 1.4-fold, 2.4-fold and 1.8-fold at 45 °C, 55 °C, and 65 °C, respectively. The optimal reaction temperature and pH for mutant E301P remained consistent with those of the wild type, at 40 °C and 10.0, respectively. The *K*_m_ of E301P was 0.22 ± 0.03 mM and the *k*_cat_ was 5.1 ± 0.28 s^−1^. Further analysis indicated that the free energies of G215A, G293A and E301P were decreased by 0.91, 0.308 and 1.1049 kcal/mol, respectively, compared to the wild-type EstSIT01. The interaction analysis revealed that the substitution of glutamic acid with proline at position 301 enhanced the hydrophobic interactions within the protein. The decreased free energies and the increased hydrophobic interactions were well correlated with the enhanced stability in these mutants.

## 1. Introduction

At the beginning of the 20th century, biotin gained significance in scientific research subsequent to Wildiers’ 1901 discovery of a crucial growth factor in yeast and malt juice, which he termed “bios” [1]. In addition to its role in preventing hair loss, biotin has been employed as a therapeutic intervention for graying hair. Furthermore, biotin functions as a coenzyme for various carboxylases and acts as a CO_2_ carrier during carboxylase reactions.

Initially, biotin was mainly extracted from costly animal and plant tissues. In 1943, Harris and his team succeeded in fully synthesizing biotin. Subsequently, in 1949, Goldberg and Stembach successfully industrialized the production of biotin through a fully synthetic process [2,3,4]. The intricacies of these methods, particularly their reliance on substantial amounts of highly toxic solvents and the low yields that impede large-scale synthesis, have prompted the development of several novel approaches for biotin synthesis. Notably, in 1970, Gerocke et al. at Roche demonstrated the high-yield production of thiolactones from lactones, which served as a cornerstone for the development of lactone-thiolactone synthetic methods. Chen’s group employed polymer-immobilized pig liver esterase (PLE) to catalyze the desymmetrization of *meso*-dimethyl ester into the (4*S*, 5*R*)-hemimethyl ester, achieving a yield of 90% and an enantiomeric excess (*e.e*.) of 91%. However, the practical application of PLE in *d*-biotin synthesis was hindered by its high cost and the relatively modest *e.e*. value [5,6].

Esterases act on short-chain fatty esters, distinguished by conserved motifs, notably Gly-X-Ser-X-Gly, and featuring a catalytic active site comprising serine residues. Typically, esterases exhibit maximal activity towards water-soluble substrates, whereas they are generally ineffective with long-chain fatty acid substrates [7]. Esterases exhibit the characteristic structural features of α/β hydrolases, including a precise arrangement of α-helices and β-sheets, as well as a conserved catalytic triad comprising Ser-His-Asp. A number of esterases possess a conserved sequence motif (GESAG) surrounding the central active-site serine residue [8]. Microbial esterases, owing to their versatile catalytic properties, rank third among the most widely used industrial biocatalysts, following amylases and proteases. They were extensively applied in pharmaceuticals, cosmetics, food processing, detergents, leather, and other industries. For instance, esterase produced by brewing microorganisms modulates the levels of various esters in wine during malolactic fermentation, significantly influencing wine aroma [9]. Furthermore, esterases are gaining significant importance in various chemical fields, including pulp and paper processing, fiber and textile treatment, detergent enhancement, fine chemicals, and biofuel production [10,11,12]. Additionally, esterases are utilized in designing biosensors for detecting or mitigating environmental pollutants, developing novel materials based on the unique properties of structural proteins, and employing sulfatase to reduce sulfate content in agar, thereby enhancing its quality [13,14,15,16].

**Figure 1:**
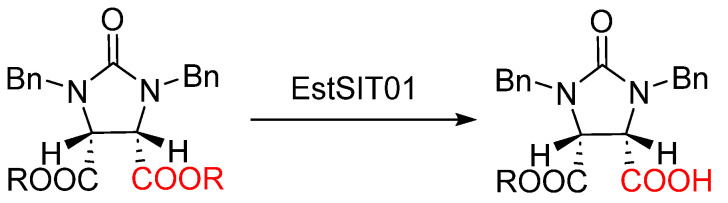
Enzymatic asymmetric hydrolysis of *meso*-diester for the production of (4*S*, 5*R*)-hemiester.

In our previous work, an esterase-producing strain, *Microbacterium* sp., capable of synthesizing biotin intermediate hemiester with a yield of up to 95% and an enantiomeric excess (*e.e.*) exceeding 99% was isolated (Figure 1). Employing genetic engineering, we successfully identified and transferred the esterase gene into *E. coli*, resulting in a recombinant strain that produces a highly selective and active esterase. The recombinant esterase exhibits significant potential for the synthesis of pharmaceutical intermediates [17].

Rational design is an effective protein engineering approach for enhancing enzyme properties through computationally predicted mutation sites. Rational design requires fewer laboratory experiments, but requires a comprehensive understanding of the intricate sequence–structure–function relationship. Several strategies can be employed to enhance protein stability via rational design. For example, rational design can enhance core stability by introducing hydrophobic interactions, either by increasing the number of hydrophobic residues or optimizing their arrangement [18,19]. Modulating charge–charge interactions, including increasing the number of surface salt bridges or strategically placing them to manipulate charge distribution, can significantly improve protein stability [20,21,22]. Additionally, the strategic design of new hydrogen bond networks can enhance protein stability [23]. Increasing proline levels or reducing glycine content can enhance thermal stability by decreasing main-chain flexibility [24,25]. Furthermore, truncation, which shortens amino acid sequences, and cyclization techniques can restrict conformational flexibility, thereby stabilizing the protein structure [26,27]. Protein design can further enhance stability by optimizing tight core packing, increasing core contacts and minimizing unfolding entropy [28,29,30].

Replacing surface amino acids, such as substituting glycine with alanine or lysine with arginine, has been shown to enhance the protein thermal stability [31]. Generally, the thermostability of proteins is influenced by the composition of their surface amino acids. Alanine and arginine contribute to increased internal hydrophobicity and stabilization of helix residues. Liu et al. successfully enhanced the thermostability of cysteine sulfinate decarboxylase through surface residue substitutions (Gly → Ala, Lys → Arg), resulting in a mutant G369A with an elevated optimal temperature [32]. Consensus mutation, a method that identifies conserved amino acid residues by aligning homologous sequences, is effective because conserved residues generally contributed more to protein folding stability than non-conserved ones [33]. The mutant T383K of glutamate decarboxylase from *Lactobacillus brevis* and variants H210N/I77L/M150C-M280C of (*R*)-selective amine transaminase from *Aspergillus terreus* exhibited significant improvements in thermostability using Consensus Finder, with *t*_1/2_ at 37 °C increasing by 1.2 times and *t*_1/2_ at 40 °C increasing by 16.6 times [34,35]. In this study, the thermostability of EstSIT01 from *Microbacterium chocolatum* SIT 101 was enhanced by integrating surface residue substitution and consensus mutation strategies. Potential substitutions within EstSIT01 were predicted using the GETAREA (http://curie.utmb.edu/getarea.html, accessed on 13 January 2023) [36] and Consensus Finder (http://kazlab.umn.edu/, accessed on 11 November 2021) [37] web-based tools. Based on experimental validation, single-point mutants that exhibited the improved thermostability were selected from the predicted candidates. Subsequently, the positive mutations were combined to generate multi-site mutants that displayed enhanced thermostability to the wild type. The positive variants were characterized and compared with the wild type, and potential mechanisms responsible for the improved thermostability were discussed.

## 2. Results

### 2.1. Selection of Sites by Surface Residue Substitution and Consensus Mutation Analysis

**Figure 2:**
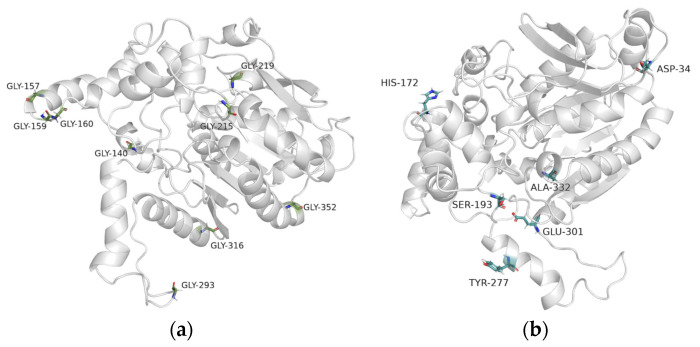
Distribution of the potential mutated residues in the structure of EstSIT01. (**a**) Glycine (green sticks) on the surface of EstSIT01 predicted by GETAREA. (**b**) Six potential mutations (blue sticks) determined by consensus analysis.

Substituting surface glycine and lysine residues with alanine and arginine is recognized as an effective strategy for enhancing protein thermal stability. The structure of EstSIT01 was modeled based on the crystal structure of aryl esterase from Burkholderia cenocepacia (PDB ID: 4X00) using ROBETTA. The structure of EstSIT01 was analyzed using GETAREA, and nine surface-exposed glycine residues were identified as potential mutation sites (Figure 2a). Being located away from the active site, the substitution of these residues is not anticipated to impact the enzyme activity. Simultaneously, the Consensus Finder, an open-source tool, was employed to predict potential amino acid substitutes based on consensus assay. After aligning 1040 homologous sequences of EstSIT01, six mutants, D34G, S193P, E301P, H172G, Y277F, and A332P were selected, with a common residue threshold exceeding 60%. As illustrated in Figure 2b, Y277F is situated in an α-helix region, while D34G, S193P, E301P, H172G, and A332P are located in the loop regions.

### 2.2. Preliminary Screening of EstSIT01 Mutants with Improved Thermal Stability or Activity

Fifteen variants, selected by GETAREA and Consensus Finder, were constructed using whole-plasmid PCR technology. The crude enzyme preparations were incubated at 55 °C for 60 min, and the residual activity of the mutants was compared to that of wild-type EstSIT01. As presented in Table 1, two variants (G215A and G316A) derived from the surface residue substitution strategy exhibited enhanced thermal stability, retaining 55.0% and 58.7% of their initial activity, respectively, whereas wild-type EstSIT01 lost 59.9% of its initial activity. In addition, the positive mutant G293A exhibited a 1.3-fold enhancement in enzymatic activity. Six variants from the consensus mutation strategy were constructed, and the results revealed that E301P and A332P exhibited improved thermal stability, with residual activities of 92.5% and 67.1%, respectively. Variants exhibiting reduced thermal stability were not further investigated in subsequent work.

Among the fifteen predicted variants, five promising mutants were identified and subsequently combined to further enhance the thermal stability and enzyme activity of EstSIT01. The combinatorial variants (E301P/G215A, E301P/G293A, E301P/G316A, A332P/G316A, E301P/A332P, and E301P/G215A/G293A) were generated and assessed for improvement in thermostability and activity. As indicated in Table 1, combination mutations were found to improve the thermal stability of the esterase. However, the combination of the most effective single-point mutant, E301P, with other single-site mutants did not always result in improved thermostability. Notably, the two-site mutant E301P/G215A exhibited a residual enzyme activity of 91.2% after incubation at 55 °C for 60 min, which was comparable to that of the single-point mutant E301P, and displayed the highest thermal stability among the two-site mutants. Conversely, the mutant E301P/G293A exhibited 179% activity compared to the wild-type enzyme while maintaining comparable stability. Further combination of G215A and G293A with E301P resulted in modest enhancements in both activity and thermostability compared with the wild type, while the additive effect was not observed. Consequently, the mutants E301P, E301P/G215A, and the triple mutant E301P/G215A/G293A were selected for subsequent investigation.

### 2.3. Thermostability and Kinetic Analysis of EstSIT01 and Its Mutants

The EstSIT01 and its mutants were purified by a Ni-affinity column and analyzed by SDS-PAGE (Figure S1). Following the purification, the three mutant enzymes (E301P, E301P/G215A, and E301P/G215A/G293A) were compared to the wild-type EstSIT01 in terms of half-life (*t*_1/2_) and enzyme kinetics. The purified enzymes were incubated at 45 °C, 55 °C and 65 °C for different times and the *t*_1/2_ value was calculated according to the residual activity. At 55 °C, the wild-type EstSIT01 exhibited a half-life (*t*_1/2_) of 33.6 min, whereas the mutants E301P, E301P/G215A and E301P/G215A/G293A exhibited significantly prolonged half-lives of 79.8 min, 68.4 min and 61.8 min, respectively, corresponding to a 2.4-, 2.0- and 1.8-fold increase, compared to EstSIT01 (Table 2). Among the mutants, E301P exhibited the highest thermal stability.

The kinetic constants of the purified enzyme were determined using biotin dimethyl ester as the substrate at 30 °C and pH 8.0. As summarized in Table 3, the specific activity of all mutants was lower than that of the wild-type enzyme. This decrease in specific activity is primarily attributed to a reduction in the catalytic constant *k*_cat_. Compared to the single-point mutant E301P, the double mutant E301P/G215A and the triple mutant E301P/G215A/G293A exhibited higher specific activities and *k*_cat_ values. Notably, the increase in *k*_cat_ for the triple mutant E301P/G215A/G293A was mainly due to the G293A mutation, which had a more significant effect than the other mutations. Despite the improvement in catalytic efficiency (*k*_cat_/*K*_m_) from single-point to multiple-point mutations, all mutants still displayed decreased catalytic efficiencies (*k*_cat_/*K*_m_) in comparison to the wild type. The decrease in catalytic efficiency primarily resulted from an increase in the *K*_m_ value, indicating a decreased affinity of the mutants for the substrate biotin dimethyl ester.

### 2.4. Enzymatic Characteristics of EstSIT01 and Its Mutant E301P

**Figure 3:**
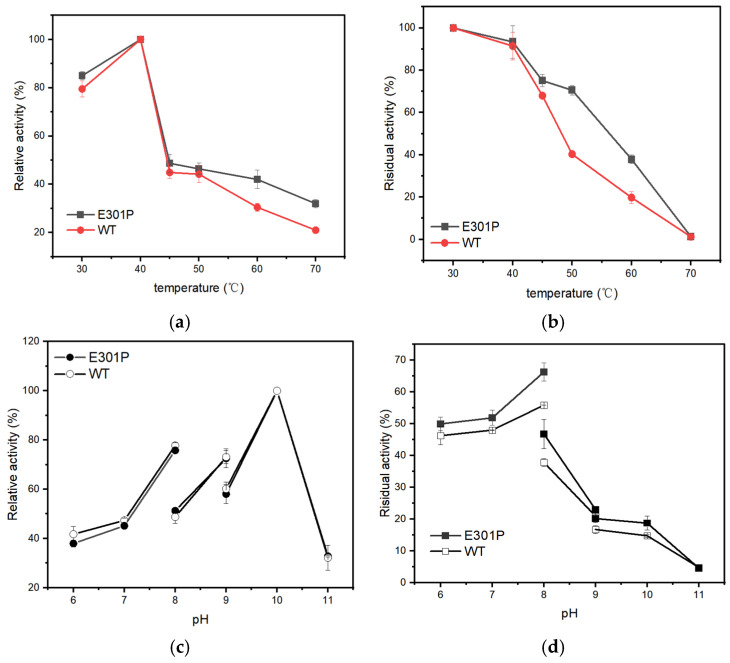
Characteristics of EstSIT01 and its variant E301P. (**a**) Effect of temperature on activity. The activities of wild-type EstSIT01 and the E301P mutant were evaluated across a range of temperatures from 30 °C to 70 °C in PBS (50 mM, pH 8.0). The maximum activities were defined as 100%, with wild-type EstSIT01 achieving 8.5 U/mg and E301P achieving 5.9 U/mg. (**b**) Thermal stability. Thermal stability was assessed by measuring the residual activity after incubating the wild-type EstSIT01 and E301P mutant at various temperatures (30 °C to 70 °C) for 60 min. The initial activity was set at 100% for both enzymes. (**c**) Effect of pH on activity. The effect of pH on enzyme activity was studied at 55 °C using different buffers: PBS for pH 6.0 to 8.0, Tris-HCl for pH 8.0 to 9.0, and Gly-NaOH for pH 9.0 to 11.0. Relative activity was expressed as a percentage of the maximum activity for each enzyme. (**d**) pH stability. pH stability was determined by monitoring residual activity after incubating the enzymes in various buffers at 55 °C for 1 h. The initial activity at each pH value was defined as 100%.

The enzymatic characteristics of purified EstSIT01 and its mutant E301P were compared. Both the wild-type and the E301P mutant exhibited an optimal reaction temperature of 40 °C (Figure 3a). The residual activity measured after incubation at various temperatures for 60 min revealed that the E301P mutant exhibited superior thermostability (Figure 3b). The mutant E301P retained approximately 70% of its activity after incubation at 50 °C for 60 min, significantly outperforming the wild-type EstSIT01, which retained only around 40% of the activity. However, at temperatures exceeding 70 °C, both the wild-type and E301P mutant were completely inactivated within 60 min, likely due to the protein structure being destroyed at higher temperatures.

Further investigation on the optimal pH indicated that both EstSIT01 and E301P displayed an optimal pH of 10.0 and exhibited similar activity trends across the pH range of 6.0 to 11.0 (Figure 3c). The residual activity, after incubating the enzymes at various pH for 60 min, indicated that E301P is more stable than EstSIT01 under pH conditions of 6.0 to 8.0 (Figure 3d). The enhanced stability of *Microbacterium* esterase could broaden its practical applications across a wide range of pH.

### 2.5. Analysis of Mutations in EstSIT01 and Their Effects on Thermal Stability and Enzyme Activity

**Figure 4:**
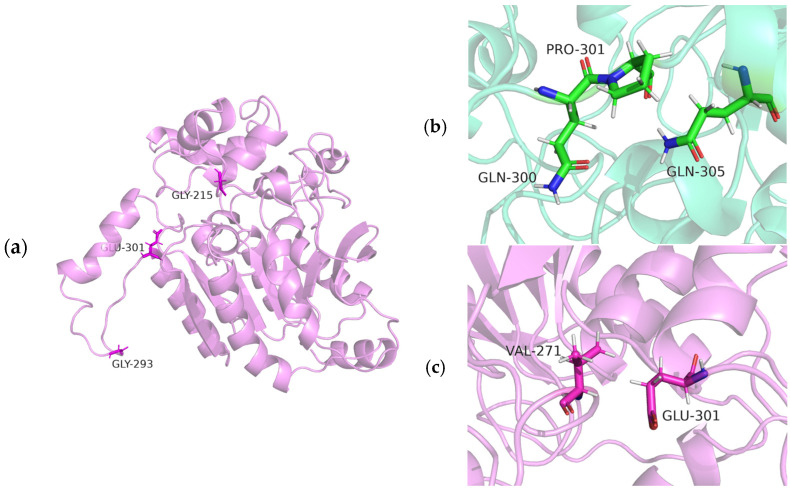
Structures of EstSIT01 (purple) and its mutants (green). (**a**) The structure of wild-type EstSIT01; (**b**) the E301P mutant formed hydrophobic interaction of Pro 301 with both Gln 300 and Gln 305; (**c**) WT exhibited hydrophobic interaction of Glu 301 with Val 271.

The modeled structure of EstSIT01 revealed the presence of a typical α/β-hydrolase fold catalytic domain (core domain) and a cap domain located above the core domain, and the three key residues, E301, G215, and G293 were presented in sticks (Figure 4a). The mutation sites at G215 and G293 were predicted by GETAREA according to the replacement of surface glycine and lysine with alanine and arginine. The differences in the calculated free energies between the mutants and the wild-type EstSIT01 (ΔΔ*G*) were calculated using the FOLDX plugin in YASARA software (version 22.5.22, YASARA Biosciences GmbH, Vienna, Austria) [38]. The free-energy changes compared to the wild type for the mutant G215A and G293A were −0.91 and −0.308 kcal/mol, respectively. The decreased free energies compared to the wild type correlate well with the enhanced stability observed in these mutants.

Among the mutants, E301, which significantly contributes to the thermal stability of EstSIT101, was predicted by Consensus Finder. The negative ΔΔG value of −1.1049 kcal/mol indicates a reduction in the free energy of the mutant, which is directly related to the protein stability. Additionally, interactions between the E301 site and adjacent residues in both the wild-type and mutant E301P were analyzed using the YASARA software. No significant changes were observed in interactions such as hydrogen bonding, with the exception of an enhanced hydrophobic interaction formed in mutant E301P. As illustrated in Figure 4b,c, P301 in the E301P mutant exhibited the hydrophobic interaction with both Q300 and Q305, in contrast to the hydrophobic interaction between E301 and V271 in wild-type EstSIT01. The substitution of glutamic acid at position 301 with proline is expected to alter the backbone structure of the enzyme. Consequently, this modification may, in turn, affect the overall conformation of the protein, potentially influencing both its stability and functionality.

Although the combination of the individual mutations always led to an additive effect, the combination of the thermostability-enhanced mutants, E301P, G293A and G215A, exhibited an antagonistic effect in our study [39]. The antagonistic effect was also observed in previous studies, and the research on engineering thermostability of the XynCDBFV discovered that combinatorial mutations within the β-sheet clusters exhibited additive effects, whereas mutations on helices or loops lead to antagonistic effects [40]. Thus, the antagonistic effect observed in our study might be attributed to the location of the E301, G215, and G293 on loops of EstSIT101 (Figure 4a). Given that high enzymatic activity is often associated with reduced stability (i.e., an activity–stability trade-off), the observed increase in activity in the double and triple mutants may result from the balance in activity–stability.

## 3. Materials and Methods

### 3.1. Chemicals, Plasmids and Strains

All chemicals utilized in this study were of analytical grade and commercially available. PrimeSTAR^®^ Max DNA Polymerase and Dpn I were purchased from Takara Biotechnology (Dalian, China). The Universal DNA Purification Kit, TIANpure Mini Plasmid Kit and DNA marker (500–7000 bp) were sourced from Tiangen Biotech Co., Ltd. (Beijing, China). The conventional range protein marker (15–130 KDa) was purchased from Sangon Biotech Co., Ltd. (Shanghai, China). *Escherichia coli* BL21(DE3) was used as the host for heterologous expression. The recombinant plasmid pET21a-*estsit01* was constructed in our previous work [41].

### 3.2. Prediction of Potential Stable Variants and Site-Directed Mutagenesis

Homology modeling of EstSIT01 was conducted using ROBETTA (https://robetta.bakerlab.org, accessed on 7 December 2024) [42], based on the crystal structure of aryl esterase from *Burkholderia cenocepacia* J2315 (PDB ID: 4X00), which has a sequence identity with EstSIT01 of 38.14%. The modeled structure was then analyzed using the GETAREA website (http://curie.utmb.edu/getarea.html, accessed on 13 January 2023) to identify surface amino acids. Glycine and lysine residues on the surface were substituted with alanine and arginine, respectively. The quality of the modeled structure was assessed using SAVES (SAVESv6.0—Structure Validation Server (ucla.edu)) [43,44,45,46]. The protein sequence in FASTA format (GenBank: WP_053548180.1) was entered into Consensus Finder (http://kazlab.umn.edu/, accessed on 11 November 2021) to locate homologous sequences and align them using Clustal X (version 2.0, University College Dublin, Dublin, Leinster, Ireland, 2010) [47]. Six potential mutation sites with a conservation threshold >60% were selected for site-directed mutagenesis. The plasmid pET21a-*estsit01* was utilized as the template to construct mutants via whole plasmid PCR [48]. The PCR reaction (50 µL) included 2× PrimeSTAR^®^ Max DNA Polymerase (25 µL), forward and reverse primers (1 µL each, 10 µM), DMSO (1 µL), plasmid DNA (1 µL), and sterile water (21 µL). The PCR program was 98 °C for 2 min (1 cycle), 98 °C for 10 s, 55 °C for 15 s, and 72 °C for 60 s (30 cycles), followed by 72 °C for 7 min (1 cycle). PCR products were digested with *Dpn* I at 37 °C for 4 h, transformed into *Escherichia coli* BL21(DE3), and cultured overnight on Luria–Bertani plates with ampicillin. Transformants were verified by DNA sequencing. Primers were synthesized by BGI (Shanghai, China), and are listed in Table S1.

### 3.3. Enzyme Expression and Purification

The optimized fermentation medium and culture methods for EstSIT01 expression have been described previously. Recombinant EstSIT01 and its variants were purified from cell lysates using BeaverBeads™ His-tag Protein Purification (BEAVER Biomedical Engineering Co., Ltd., Suzhou, China), following the manufacturer’s guidelines. Cells were harvested, resuspended in binding buffer (20 mM NaH_2_PO_4_, 500 mM NaCl, 50 mM imidazole, pH 7.4), and then sonicated on ice. The *E. coli* cell lysates were centrifuged at 15,000× *g* for 30 min at 4 °C. The obtained supernatants (cell-free extracts) were used as crude enzyme solution for the preliminary test or for subsequent purification.

His-tagged target proteins were adsorbed onto magnetic beads and eluted with elution buffer (20 mM NaH_2_PO_4_, 500 mM NaCl, 250 mM imidazole, pH 7.4). Imidazole was subsequently removed by ultrafiltration against PBS buffer (50 mM, pH 8.0) using a 10 kDa cutoff membrane (EMD Millipore, Billerica, MA, USA). Protein purity was assessed by 12% SDS-PAGE, and protein concentration was determined using the Bradford method [49], with bovine serum albumin as the standard.

### 3.4. Enzyme Activity Assay and Kinetic Analysis

The enzyme activity was measured in a reaction system containing 50 mM PBS buffer (pH 8.0), 10 mM biotin dimethyl ester, and appropriately diluted enzyme solution, incubated at 30 °C for 10 min. The reaction was halted by adding 1 mL of anhydrous methanol. The concentration of biotin monomethyl ester in the sample was determined by HPLC. One unit of EstSIT01 activity (U) is defined as the amount of enzyme that produces 1 µmol of biotin monomethyl ester per minute under standard conditions (pH 8.0, 30 °C). To determine kinetic parameters, enzyme activity was measured at biotin dimethyl ester concentrations ranging from 0.1 to 10 mM. Kinetic values were calculated by fitting the Michaelis–Menten plot using nonlinear regression with GraphPad Prism software (version 8.0, GraphPad Software Inc., San Diego, CA, USA, 1995).

### 3.5. Thermal Stability Assay

The thermal stability of EstSIT01 was evaluated by measuring the half-life (*t*_1/2_) of the enzyme at different temperatures. Purified wild-type enzyme and mutants (0.3 mg/mL) were incubated at 45 °C, 55 °C, and 65 °C for varying periods. At specified intervals, samples were removed, placed on ice for 10 min, and then residual enzyme activity was measured as described previously.

The first-order inactivation rate constant (*k*_d_) was estimated by linear regression of ln (residual activity) against incubation time, with *t*_1/2_ calculated as ln2/*k*_d_.

### 3.6. Effect of Temperature and pH Inhibition on Enzyme Activity

To determine the optimal temperature, enzyme activity was assessed in PBS buffer (50 mM, pH 8.0) across a temperature range of 30 °C to 70 °C. Relative activity was calculated as the ratio of enzyme activity at different temperatures to its maximum activity (wild-type: 8.5 U/mg, E301P: 5.9 U/mg). For thermal inactivation studies, purified enzyme (0.3 mg/mL) was incubated at temperatures ranging from 30 °C to 70 °C for 60 min, with residual activity measured as a percentage of the initial activity. Experiments were performed in triplicate. To determine optimal pH, reactions were carried out at 30 °C in various buffers, with pH values ranging from 6.0 to 11.0. Relative activity was expressed as a percentage of maximum activity. pH stability was analyzed by measuring residual activity after incubation at 55 °C for 1 h in the corresponding buffer, with the activities of wild-type EstSIT01 and E301P defined as 100%. Average values from three independent measurements are reported.

## 4. Conclusions

The rational-design approach was successfully employed to enhance the thermal stability of EstSIT01 from *Microbacterium chocolatum* in this study. The potential mutation sites were identified by substituting surface amino acids (Gly → Ala, Lys → Arg) and employing consensus mutations. Among the 15 predicted mutants, the E301P mutant exhibited a notable 2.4-fold increase in half-life at 55 °C compared to the wild-type, representing the most significant improvement. Combining mutations into double- and triple-point variants, such as E301P/G215A, and G293A/E301P/G215A, did not lead to further enhancement in stability, but did boost enzyme activity. This approach demonstrates a strategic method for optimizing enzyme performance in industrial applications, especially at higher temperatures.

## Figures and Tables

**Table 1:** Comparison of enzyme activity and residual activity of EstSIT01 with positive mutants.

	Enzyme	Relative Activity (%) ^a^	Residual Activity (%) ^b^
	EstSIT01	100.0 ± 8.4	40.1 ± 3.3
Surface residue replacement	G215A	109.5 ± 9.9	55.0 ± 3.8
	G316A	64.5 ± 3.3	58.7 ± 5.5
	G293A	132.7 ± 7.4	44.5 ± 3.8
Consensus mutagenesis	A332P	63.3 ± 1.3	67.1 ± 3.8
	E301P	100.4 ± 3.2	92.6 ± 1.6
Combinatorial mutagenesis	E301P/G293A	179.1 ± 3.2	42.5 ± 2.5
	E301P/G316A	67.2 ± 3.4	41.4 ± 1.0
	A332P/G316A	68.3 ± 9.2	80 ± 0.1
	E301P/A332P	84.6 ± 5.6	56.2 ± 3.2
	E301P/G215A	84.2 ± 6.1	91.2 ± 1.0
	E301P/G215A/G293A	110.0 ± 8.4	69.8 ± 1.2

**^a^** Enzyme activity was measured at 30 °C and pH 8.0 (0.05 M PBS) for 10 min, and the activity of wild-type EstSIT01 was set as 100%. ^b^ The residual activity of the crude enzyme was determined after incubation at 55 °C for 60 min, and the initial activity of each mutant was defined as 100%, respectively.

**Table 2:** Heat-inactivated half-lives of EstSIT01 and its mutants.

Enzymes	*t*_1/2_ (min) ^a^		
	45 °C	55 °C	65 °C
Wild-type	126.0	33.6	15.6
E301P	180.6	79.8	28.8
E301P/G215A	143.4	68.4	27.0
E301P/G215A/G293A	133.8	61.8	24.6

^a^ The enzyme of 0.3 mg/mL was incubated at various temperature. Samples were collected at various time intervals and the residual activities was measured at pH 8.0 (0.05 M PBS) and 30 °C.

**Table 3:** Kinetic parameters and specific activities of EstSIT01 and its mutants.

Enzyme	*K*_m_ (mM)	*k*_cat_ (s^−1^)	*k*_cat_/*K*_m_ (mM^−1^·s^−1^) ^a^	Specific activity (U·mg^−1^) ^b^
Wild-type	0.15 ± 0.02	7.8 ± 0.31	51.9	8.5 ± 0.21
E301P	0.22 ± 0.03	5.1 ± 0.28	23.2	5.9 ± 0.11
E301P/G215A	0.21 ± 0.04	5.4 ± 0.37	25.6	6.5 ± 0.32
E301P/G215A/G293A	0.18 ± 0.03	6.3 ± 0.39	34.9	7.5 ± 0.29

^a^ The kinetic parameters were measured at a pH of 8.0 in 0.05 M PBS at 30 °C, with biotin dimethyl ester concentrations varying from 0.1 to 10 mM. ^b^ The specific activity was assayed at pH 8.0, 30 °C, in 0.05 M PBS, with a substrate concentration of 10 mM.

## Data Availability

Data are contained within the article.

## Supplementary Materials

The following supporting information can be downloaded at: https://www.mdpi.com/article/10.3390/molecules29245839/s1, Figure S1: SDS-PAGE assay of purified EstSIT01 and its mutants; Table S1: Primers for site-directed mutagenesis.

## References

[1] Wildiers E. (1901). A New Substance Indispensable to the Development of Yeast. J. La Cellule.

[2] Goldberg M.W., Stembach L.H. (1949). Synthesis of Biotin. U.S. Patent.

[3] Goldberg M.W., Stembach L.H. (1949). Synthesis of Biotin. U.S. Patent.

[4] Goldberg M.W., Sternbach L.H. (1949). Debenzylation of Benzylated Imidazolido-Thiophane Compounds. U.S. Patent.

[5] Chen X.X., Xiong F., Fu H., Liu Z.Q., Chen F.E. (2011). Synthetic studies on (+)-biotin, part 15: A chiral squaramide-mediated enantioselective alcoholysis approach toward the total synthesis of (+)-biotin. J. Chem. Pharm. Bull..

[6] Chen F.E., Chen X.X., Dai H.F., Kuang Y.Y., Xie B., Zhao J.F. (2005). Synthetic studies on *d*-Biotin, Part 8:[1] An Efficient chemoenzymatic approach to the asymmetric total synthesis of *d*-Biotin via a polymer-supported PLE-mediated desymmetrization of *meso*-symmetric dicarboaylic esters. J. Adv. Synth. Catal..

[7] Uwe T. (2002). Bornscheuer, Microbial carboxyl esterases: Classification, properties and application in biocatalysis. FEMS Microbiol. Rev..

[8] Drabløs F., Petersen S.B. (1997). Identification of conserved residues in family of esterase and lipase sequences. Methods Enzymol..

[9] Darsonval M., Alexandre H., Grandvalet C. (2016). Genetically engineered *Oenococcus oeni* strains to highlight the impact of estA2 and estA7 esterase genes on wine ester profile. Food Microbiol..

[10] Clarke N. (2010). Protein engineering for bioenergy and biomass-based chemicals. Curr. Opin. Struct. Biol..

[11] Carvalho C.C. (2011). Enzymatic and whole cell catalysis: Finding new strategies for old processes. Biotechnol. Adv..

[12] Tang W.L., Zhao H. (2009). Industrial biotechnology: Tools and applications. Biotechnol. J..

[13] Alcalde M., Ferrer M., Plou F.J., Ballesteros A. (2006). Environmental biocatalysis: From remediation with enzymes to novel green processes. Trends Biotechnol..

[14] Zhu Y., Liu H., Qiao C., Li L., Jiang Z., Xiao A., Ni H. (2017). Characterization of an arylsulfatase from a mutant library of *Pseudoalteromonas carrageenovora* arylsulfatase. Int. J. Biol. Macromol..

[15] Tan X., Yu C., Tang J., Wu W., Yang Q., Hou X. (2024). Progress in Nanomaterials-Based Enzyme and Aptamer Biosensor for the Detection of Organophosphorus Pesticides. Crit. Rev. Anal. Chem..

[16] Gong C., Fan Y., Zhao H. (2022). Recent advances and perspectives of enzyme-based optical biosensing for organophosphorus pesticides detection. Talanta.

[17] Li X.J., Yu H.R., Liu S.L., Ma B.D., Wu X.M., Zheng X.S., Xu Y. (2024). Discovery, characterization and mechanism of a *Microbacterium* esterase for key *d*-biotin chiral intermediate synthesis. Bioresour. Bioprocess..

[18] Pace C.N., Fu H., Fryar K.L., Landua J., Trevino S.R., Shirley B.A., Hendricks M.M., Iimura S., Gajiwala K., Scholtz J.M. (2011). Contribution of hydrophobic interactions to protein stability. J. Mol. Biol..

[19] Strub C., Alies C., Lougarre A., Ladurantie C., Czaplicki J., Fournier D. (2004). Mutation of exposed hydrophobic amino acids to arginine to increase protein stability. BMC Biochem..

[20] Wijma H.J., Floor R.J., Jekel P.A., Baker D., Marrink S.J., Janssen D.B. (2014). Computationally designed libraries for rapid enzyme stabilization. J. Protein Eng. Des. Sel..

[21] Contessoto V.G., de Oliveira V.M., Fernandes B.R., Slade G.G., Leite V.B.P. (2018). TKSA-MC: A web server for rational mutation through the optimization of protein charge interactions. Proteins.

[22] Lee C.W., Wang H.J., Hwang J.K., Tseng C.P. (2014). Protein thermal stability enhancement by designing salt bridges: A combined computational and experimental study. PLoS ONE.

[23] Livesay D.R., Huynh D.H., Dallakyan S., Jacobs D.J. (2008). Hydrogen bond networks determine emergent mechanical and thermodynamic properties across a protein family. Chem. Cent. J..

[24] Argos P., Rossman M.G., Grau U.M., Zuber H., Frank G., Tratschin J.D. (1979). Thermal stability and protein structure. Biochemistry.

[25] Nicholson H., Tronrud D.E., Becktel W.J., Matthews B.W. (1992). Analysis of the effectiveness of proline substitutions and glycine replacements in increasing the stability of phage T4 lysozyme. Biopolymers.

[26] Liu L., Yu H., Du K., Wang Z., Gan Y., Huang H. (2018). Enhanced trypsin thermostability in Pichia pastoris through truncating the flexible region. Microb. Cell Fact..

[27] Si M., Xu Q., Jiang L., Huang H. (2016). SpyTag/SpyCatcher cyclization enhances the thermostability of firefly luciferase. PLoS ONE.

[28] Matthews B.W., Nicholson H., Becktel W.J. (1987). Enhanced protein thermostability from site-directed mutations that decrease the entropy of unfolding. J. Proc. Natl. Acad. Sci. USA.

[29] Gonzalez N.A., Li B.A., McCully M.E. (2022). The stability and dynamics of computationally designed proteins. Protein Eng. Des. Sel..

[30] Wang R., Wang S., Xu Y., Yu X.W. (2020). Enhancing the thermostability of *Rhizopuschinensis* lipase by rational design and MD simulations. Int. J. Biol. Macromol. Macromol..

[31] Ye S.S., Zhou L., Zhou Z.M. (2016). Thermal stability improvement for phenylalanine hydroxylase by site-directed mutagenesis. Chin. J. Biotech..

[32] Liu Z., Zheng W., Ye W., Wang C., Gao Y., Cui W., Zhou Z. (2019). Characterization of cysteine sulfinic acid decarboxylase from *Tribolium castaneum* and its application in the production of β-alanine. Appl. Microbiol. Biotechnol..

[33] Porebski B.T., Buckle A.M. (2016). Consensus protein design. Protein. Eng. Des. Sel..

[34] Hua Y., Lyu C., Liu C., Wang H., Hu S., Zhao W., Mei J., Huang J., Mei L. (2020). Improving the Thermostability of Glutamate Decarboxylase from *Lactobacillus brevis* by Consensus Mutagenesis. Appl. Biochem. Biotechnol..

[35] Xie D.F., Yang J.X., Lv C.J., Mei J.Q., Wang H.P., Hu S., Zhao W.R., Cao J.R., Tu J.L., Huang J. (2019). Construction of stabilized (*R*)-selective amine transaminase from *Aspergillus terreus* by consensus mutagenesis. J. Biotechnol..

[36] Fraczkiewicz R., Braun W. (1998). Exact and Efficient Analytical Calculation of the Accessible Surface Areas and Their Gradients for Macromolecules. J. Comp. Chem..

[37] Jones B.J., Kan C.N.E., Luo C., Kazlauskas R.J. (2020). Consensus Finder web tool to predict stabilizing substitutions in proteins. Meth. Enzymol..

[38] Schymkowitz J., Borg J., Stricher F., Nys R., Rousseau F., Serrano L. (2005). The FoldX web server: An online force field. Nucleic Acids. Res..

[39] Bednar D., Beerens K., Sebestova E., Bendl J., Khare S., Chaloupkova R., Prokop Z., Brezovsky J., Baker D., Damborsky J. (2015). FireProt: Energy- and Evolution-Based Computational Design of Thermostable Multiple-Point Mutants. PLoS Comput. Biol..

[40] Han N., Ma Y., Mu Y., Tang X., Li J., Huang Z. (2019). Enhancing thermal tolerance of a fungal GH11 xylanase guided by B-factor analysis and multiple sequence alignment. Enzym. Microb. Technol..

[41] Wei K., Wu X., Ma B., Li Z., Xu Y. (2022). Facile immobilization of his-tagged Microbacterial esterase on Ni-SBA-15 with enhanced stability for efficient synthesis of key chiral intermediate of *d*-biotin. Bioprocess. Biosyst. Eng..

[42] Kim D.E., Chivian D., Baker D. (2004). Protein structure prediction and analysis using the Robetta server. Nucleic Acids Res..

[43] Laskowski R.A., MacArthur M.W., Moss D.S. (1993). PROCHECK: A program to check the stereochemical quality of protein structures. J. Appl. Crystallogr..

[44] Bowie J.U., Luthy R., Eisenberg D. (1991). A method to identify protein sequences that fold into a known three-dimensional structure. Science.

[45] Colovos C., Yeates T.O. (1993). Verification of protein structures: Patterns of nonbonded atomic interactions. Protein Sci..

[46] Pontius J., Richelle J., Wodak S.J. (1996). Deviations from standard atomic volumes as a quality measure for protein crystal structures. J. Mol. Biol..

[47] Larkin M.A., Blackshields G., Brown N.P. (2007). Clustal W and Clustal X version 2.0. Bioinformatics..

[48] Ho S.N., Hunt H.D., Horton R.M., Pullen J.K., Pease L.R. (1989). Site-directed mutagenesis by overlap extension using the polymerase chain reaction. Gene.

[49] Bradford M.M. (1976). A rapid and sensitive method for the quantitation of microgram quantities of protein utilizing the principle of protein-dye binding. Anal. Biochem..

