# Supplementary material for: Enhancement of the Thermostability of Microbacterium Esterase by Combinatorial Rational Design
Source: Molecules. 2024 Dec 11;29(24):5839. doi: 10.3390/molecules29245839 (PMC11676285; doi:10.3390/molecules29245839)
Supplement: Supplementary file 1 [file molecules-29-05839-s001.zip › molecules-3341129-supplementary.pdf]

Supplementary Information

Enhancement of the thermostability of *Microbacterium* esterase by combinatorial rational design

Wenyu Peng[1], Xiaomei Wu[1], Wenlong Liu[2], Baodi Ma[1*] and Yi Xu[1*]

1 School of Chemical and Environmental Engineering, Shanghai Institute of Technology, Shanghai 201418, China; 196061523@mail.sit.edu.cn (W. P.); wuxiaomei@sit.edu.cn (X. W.)

2 Shandong Lonct Enzymes Co., Ltd, Linyi 276400, China; 13573926636@163.com (W. L.)

* Correspondence: xuyi@sit.edu.cn (Y. X.), mbd2966@sit.edu.cn (B. M.); Tel.: +86-21-60873024

Table of Contents

Figure S1. SDS-PAGE assay of purified EstSIT01 and its mutants 1

Table S1. Primers for site-directed mutangenesis 2

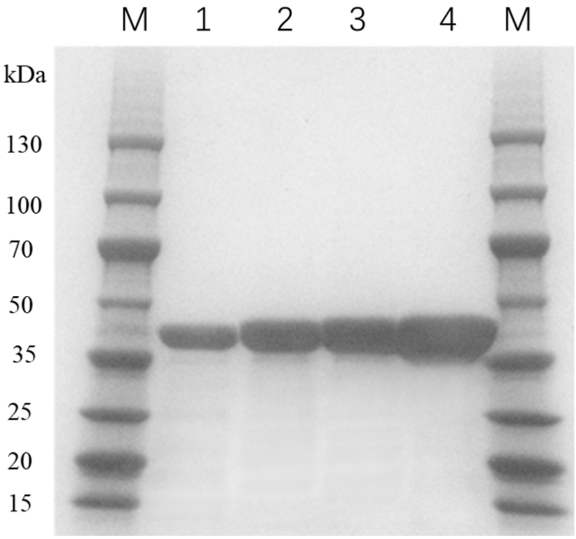

Figure S1. SDS-PAGE assay of purified EstSIT01 and its mutants. M: Marker; Lane 1: Wild-type; EstSIT01; Lane 2: mutant E301P; Lane 3: mutant E301P/G215A; Lane 4: E301P/G215A/G293A.

Table S1. Primers for site-directed mutangenesis

Primers	Sequences (5'→3')	Length (bp)	T_m (°C)
E301P-F	GCCCGCGCAGCCGATGGTGTCGCAGACC	28	74.22
E301P-R	GCGACACCATCGGCTGCGCGGGCGCGAC	28	75.69
A332P-F	GCCGTCGAGGGCGCGGGCCACTCCCCACACCTGGAGCG	38	81.48
A332P-R	GAACACCGCGGGACGCTCCAGGTGTGGGGAGTGGCCCG	38	79.32
H172G-F	GACTTCATCCAGCGCCTCATCGACGGAGACACCTCCG	37	74.17
H172G-R	GGTCTGCGCATCGTCGGAGGTGTCTCCGTCGATGAGG	37	75.28
Y277F-F	CGCGTCGTTCTTCGACCTCAACTACCTC	28	66.90
Y277F-R	AGTTGAGGTCGAAGAACGACGCGTCGGA	28	66.90
S193P-F	CTACGTCGCCCCGGACTACACCACCGAC	28	71.29
S193P-R	TGGTGTAGTCCGGGGCGACGTAGCCGGC	28	72.76
D34G-F	GACCCCGCCCGGACGCACGGTCGTG	25	73.70
D34G-R	CGACCGTGCGTCCGGGCGGGGTCTG	25	73.70
G140A-F	CGGTGTCGCCCTACGGCTTCGCAGGCACCCGCC	33	77.78
G140A-R	GAGCCGTCACGGCGGGTGCCTGCGAAGCCGTAG	33	76.54
G157A-F	CGACGATGCCGGCTGCGCAGGCGGCGGTGC	30	77.70
G157A-R	GGTTCGCACCGCCGCCTGCGCAGCCGGCAT	30	76.33
G159A-F	GATGCCGGCTGCGGTGGCGCAGGTGCGAACC	31	75.96
G159A-R	GAAGTCGGGGTTCGCACCTGCGCCACCGCAG	31	74.64
G160A-F	CGGCTGCGGTGGCGGCGCAGCGAACCCCGACT	32	78.18
G160A-R	TGAAGTCGGGGTTCGCTGCGCCGCCACCGCAG	32	75.62
G215A-F	CCACGTCCACCGCCGACGCAAACTACC	27	70.01
G215A-R	GCATCGCCGGGGTAGTTTGCGTCGGCG	27	71.53
G219A-F	CCACCGCCGACGGAAACTACCCCGCAGATGCGGTGCC	37	77.49
G219A-R	CAGTTGTCGCTCGGCACCGCATCTGCGGGGTAGTTTC	37	74.17
G293A-F	CGGCTGGCCCGCAGAAGACGTCGCGCCCGCGCAGG	35	80.56
G293A-R	CGACGTCTTCTGCGGGCCAGCCGGGGACGATGCCC	35	78.21
G316A-F	CTACGCTGCGGCAGGCGGAACGGTCACCGAGGTCGCC	37	78.60
G316A-R	CCGTTCCGCCTGCCGCAGCGTAGCGGCCGAGGACATC	37	78.60
G317A-F	GTCCTCGGCCGCTACGCTGCGGGCGCAGGAACGGTCACCG	40	80.65
G317A-R	GACGGCGACCTCGGTGACCGTTCCTGCGCCCGCAGCGTAG	40	79.62
G352A-F	CGGCTACGTCGCAGCGGCGGCCGACCCCGCCCCGC	35	82.90
G352A-R	CGGCCGCCGCTGCGACGTAGCCGATGATCTCGAGC	35	77.04
